# Development and validation of MRI-based radiomics model for clinical symptom stratification of extrinsic adenomyosis

**DOI:** 10.1080/07853890.2025.2534521

**Published:** 2025-07-25

**Authors:** Man Sun, Jianzhang Wang, Ping Xu, Libo Zhu, Gen Zou, Shuyi Chen, Yuanmeng Liu, Xinmei Zhang

**Affiliations:** Department of Gynecology, Women’s Hospital, Zhejiang University School of Medicine, Hangzhou, China

**Keywords:** Adenomyosis, heterogeneity, MRI, radiomics, symptom

## Abstract

**Background:**

Extrinsic adenomyosis exhibits heterogeneous clinical symptoms, with pain being more commonly reported. The relationship between magnetic resonance imaging (MRI) feature and symptom remains unclear.

**Objective:**

To evaluate the performance of MRI radiomics model for differentiating symptom heterogeneity of extrinsic adenomyosis, pain, abnormal uterine bleeding (AUB), infertility, and no symptom.

**Materials and methods:**

This retrospective analysis included 405 patients with MRI-diagnosed extrinsic adenomyosis (January 2020-July 2022), randomly split 7:3 into training and test cohorts. Radiomic features were extracted from MRI-T2 image. Random forest algorithm was used to select the key radiomics features of different symptoms and develop the radiomic model by support vector machine algorithm. Multivariable logistic regression assessed clinical characteristics. A combined radiomics-clinical nomogram was created for symptom stratification.

**Results:**

In total 405 patients presented with 496 clinical symptoms. In the training and test cohorts, radiomics models achieved areas under the curve (AUCs) of 0.73/0.72 (pain), 0.82/0.76 (AUB), 0.84/0.80 (infertility), and 0.80/0.71 (no symptom). The multi-signature model (radiomic + clinical features) showed improved performance, with the nomogram demonstrating good stratification ability: AUCs of 0.78/0.78 (pain), 0.87/0.85 (AUB), 0.89/0.88 (infertility), and 0.84/0.81 (no symptom) in the training/test cohort.

**Conclusion:**

We identified the correlation between key radiomic features and clinical symptom of extrinsic adenomyosis. The machine learning-based MRI radiomics models have potential for symptom stratification of extrinsic adenomyosis and may potentially reduce unnecessary treatment.

## Introduction

Adenomyosis is a gynecological uterine disease, presented as the pathological invasion of endometrial glands and stroma in myometrium. The clinical symptom of adenomyosis is complex, multifactorial, with heterogeneous presentation, including pain, abnormal uterine bleeding, infertility and some patient with no symptom [1]. Adenomyosis lesions of the inner and outer myometrium are associated with different clinical features. Pelvic pain and dysmenorrhea occurred more in the women exhibiting external adenomyosis [2]. However, there is no consensus on clinical symptom and MRI phenotype of adenomyosis. It is important to investigate how imaging parameters features relate to clinical symptom.

Nowadays, adenomyosis can be increasingly diagnosed by MRI that considered to be a non-invasive tool. MRI have become widely accessible in adenomyosis diagnosis, that make a shift into a preoperative imaging-centered diagnosis instead of a postoperative pathology-centered diagnosis (specimen resected from hysterectomy) [3]. In most studies, MRI of diagnosing in adenomyosis achieved a better specificity and sensitivity. Although, traditional imaging features of adenomyosis in MRI performed in initial screening and clinical symptomatology stratification, a recent data analysis showed that there are image signs they remain non-specific [4]. The previous study showed the presence and/or worsening of painful symptoms were more frequently reported in patients with disease progression [5]. Thus, given the importance of capturing lesion heterogeneity, MRI holds the potential for improving the clinical symptomatology stratification of adenomyosis.

Radiomics quantified the image-tracked entire lesions and provided more high-throughput quantitative information about lesion heterogeneity, such as morphology, intensity, and texture [6]. Radiomics features and model demonstrate more comprehensive information and reflect its biological behavior [7]. A recent study found that the use of MRI radiomics can predict the long-term outcomes of magnetic resonance-guided focused ultrasound ablation therapy in patients with adenomyosis [8]. Radiomics can detect latent information invisible to the naked eye and identify impending clinical symptoms. By identifying patients at high risk of infertility, personalized fertility guidance can be provided in advance, relieving psychological stress and saving time. Early detection of AUB risk helps avoid complications like anemia and reduces medical resource waste. Predicting the adenomyosis lesion status will help clinicians develop more personalized treatment strategies.

To classify adenomyosis based on symptoms is particularly important for clinical diagnosis and medical practice allowing for personalized and precise diagnosis and treatment. The objective of this study was to explore the relationship between clinical symptom of extrinsic adenomyosis and MRI radiomics features in order to construct radiomics model for different clinical symptom subtypes. The latter radiomics model combined with clinical characteristics nomogram was utilized to assess the potential stratification of extrinsic adenomyosis. This study was able to efficiently assess the clinical status of extrinsic adenomyosis.

## Materials and methods

### Patients

A total of 443 extrinsic adenomyosis patients who underwent MRI from women’s hospital school of medicine Zhejiang university (From January 2020 to July 2022) were considered for inclusion in this study. Extrinsic adenomyosis can be defined according to kishi’s classification [9]. All images were re-read by two specialists of adenomyosis to exclude controversial images after radiological diagnosis. Inclusion criteria were as follows: (i) age 18–50 years old, (ii) adenomyosis patients who underwent pelvic MRI examination, (iii) complete clinical data. Exclusion criteria in our study were as follows: gynecologic malignant tumors, menopause, pregnancy, large uterine myomas (single diameter > 4 cm) uterine malformations, uterine adhesion, male factor infertility and lack of clinical data were excluded. Finally, 405 of 443 primary screening were included. This study was approved by the institutional ethics review board of Women’s Hospital School of Medicine, Zhejiang University and adhered to the principles of the Helsinki Declaration (ethics approval No. IRB-20220339-R), and written informed consent was obtained from all patients. Readily available clinical characteristics were documented, including age, height, weight, BMI, gravidity, parity, mean age at menarche, mean duration cycle, mean length of menstruations, regular menstrual cycle, mean length of menstruations, regular menstrual cycle, endometriotic cyst, deep infiltrating endometriosis, uterine myoma, history of miscarriage, previous uterine surgery, CA125, junctional zone size, mean myometrium, junctional zone/myometrium ratio.

We used machine learning models to identify the symptoms of dysmenorrhea, AUB, infertility and no symptom in extrinsic adenomyosis patients. Adenomyosis associated with pain include dysmenorrhea, dyspareunia, chronic pelvic pain, bladder and gastrointestinal pain symptoms, such as dysuria and dyschezia. Abnormal uterine bleeding (AUB) means bleeding from the uterine cavity that differs from normal menstruation in aspects like cycle frequency, regularity, duration, and menstrual flow amount. Adenomyosis often shows as heavy menstrual bleeding, prolonged or irregular bleeding, intermenstrual bleeding, and pre - or postmenstrual spotting. Infertility is clinically defined as the failure to achieve a pregnancy following 12 months of regular, unprotected sexual intercourse, with "regular" typically denoting coitus occurring at least 2–3 times per week. For example, if a patient reported both AUB and pain, then this patient was included in both the pain positive group and the AUB positive group. Therefore, 405 patients in this study presented with 496 kinds of symptoms. The dataset was split into a training cohort (*n* = 284), and a testing cohort (*n* = 121) with a 7:3 ratio using a stratified randomization method to keep the similar ratio between negative and positive samples in two cohort. The "positive cohort" refers to the group of patients who exhibit the symptom of interest. The positive cohort consists of patients who reported pain, AUB, infertility. Conversely, the "negative cohort" represents the group of patients who did not report the symptom.

### MRI image acquisition

Extrinsic adenomyosis patients were performed at a 1.5-T MRI system (GE Healthcare) before treatment. The imaging data we utilized were obtained prior to the initiation of any treatment, including hormonal therapy. Signal transmission was accomplished using body coils, and signal reception was with an 8-channel cardiac coil. T2-weighted pelvic MRI images were acquired in 3-plane localization of axial, sagittal, and coronal planes. Imaging parameters were following as: slice thickness = 5.6 mm, and spacing = 1 mm, matrix = 512 × 512, repetition time/echo time = 3000–6000/90 ms, field of view = 100 mm.

### Lesion segmentation

All MRI images were collected from radiological department’s picture system. Digital imaging and Communications in Medicine images (DICOM) of the portal venous phase from the picture archiving and communication system were retrieved for radiomic analysis. All image processing and standardization follows the image biomarker standardization initiative (IBSI) [10]. In this study, the adenomyosis lesion region of interest (ROI) for each patient were delineated by 3D slicer software (https://www.slicer. org/). ROIs were placed on all slices that contained the whole adenomyosis lesion in T2-weighted images. Two readers (Jian Zhang wang, Xinmei Zhang with 7, 20 years of experience in adenomyosis imaging, respectively) were blinded and independently reviewed all images to draw ROIs. The traditional MRI radiologic feature were evaluated as following: junctional zone size, mean myometrium, junctional zone/myometrium ratio. Additionally, to evaluate the feature stability, two readers repeated the ROI drawing at least 1 month. Intraclass correlation coefficients (ICCs) were utilized to evaluate the intra- and interobserver agreement in terms of feature extraction. ICC higher than 0.8 was considered a suitable marker. The whole ROIs were completed to promise a better consistency.

### Radiomics analysis and model construction

Radiomics features were extracted using the IBSI-compliant, Pyradiomics 3.0 python package, implemented in Python 3.7. The types of radiomics features included shape, first order, textural, wavelet. In total 1474 radiomics feature were extracted for each lesion. Next, the random forest (RF) algorithm was trained to determine the predictive power of different symptoms. Then these tops features identified by RF were applied for the model construction of the signature *via* the support vector machine (SVM) algorithm. To account for class imbalance, the number of selected features was obtained through the tenfold cross-validation procedure.

### Clinical–radiomic nomogram

A univariate analysis was used to select the clinical variables in different symptom group. Then the significant clinical variables were used to develop clinical signatures for different symptom prediction. Clinical signatures and radiomic features also not highly correlated with each other were retained for subsequent multivariable regression. Finally, a clinical–radiomic nomogram was validated that could distinguish symptom.

### Statistical analysis

The Kolmogorov–Smirnov test assessed the normality of distribution before statistical comparisons [11]. Continuous variables were analyzed using Student’s *t* test and Wilcoxon’s test. For categorical data without order, Pearson’s chi-square test and Fisher’s exact test were applied. All statistical tests were conducted as two-tailed, with *p* < 0.05 considered significant. All data processing and statistical analyses were performed with Python (version 3.7; https://www.python.org/) and R software (version 4.1.0; https://www.r-project.org)

## Results

### Patients and clinical characteristics

**Figure 1.**
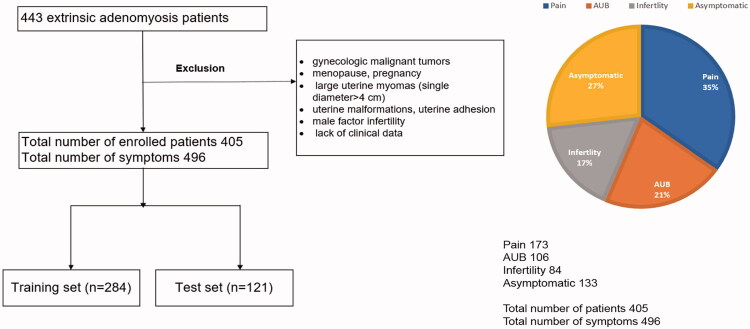
Flowchart of patient selection and clinical symptom distribution.

**Figure 2.**
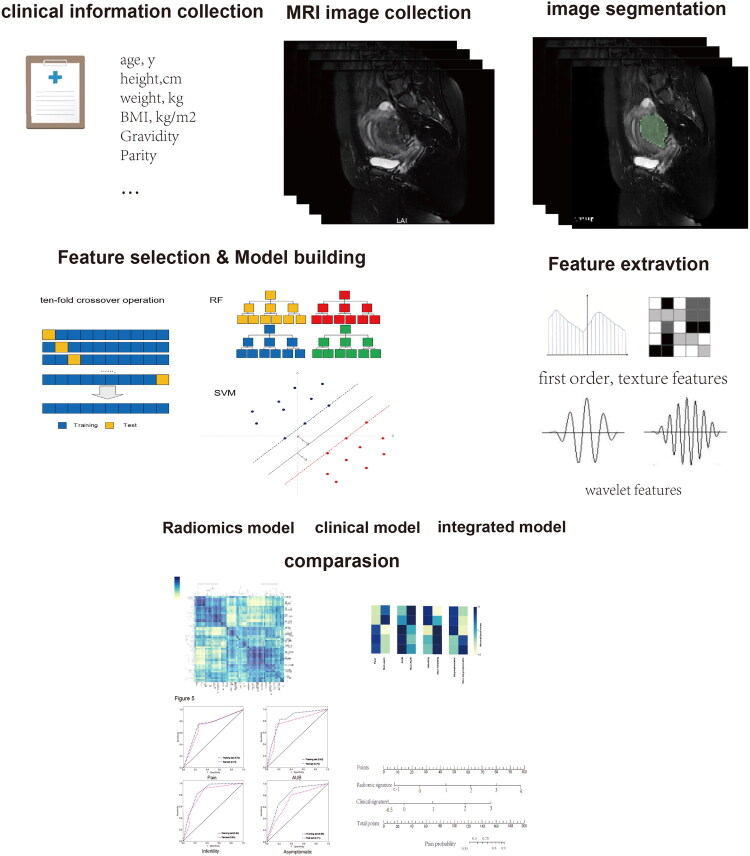
Work flow of radiomics and model construction, evaluation.

**Figure 3.**
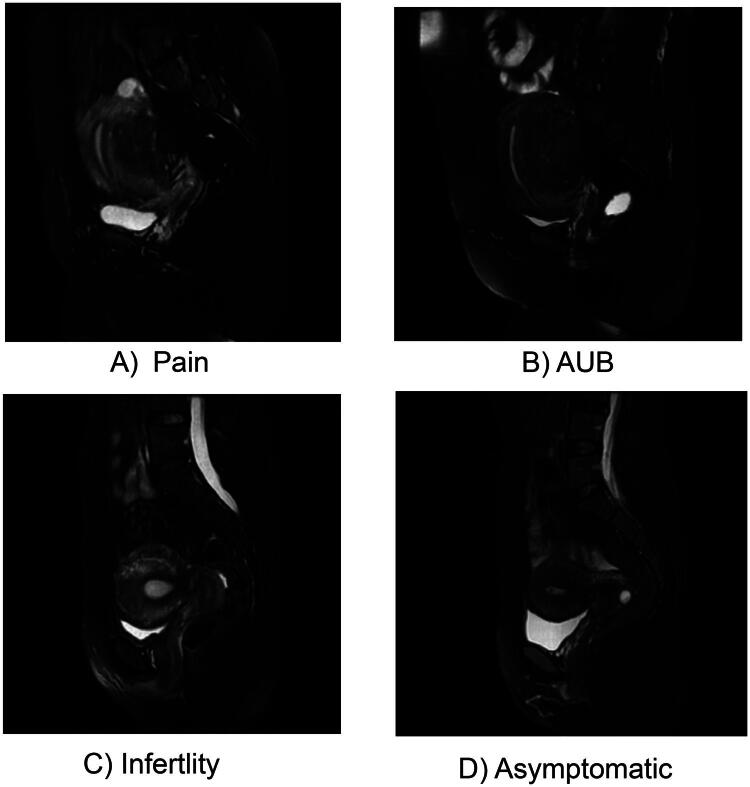
MRI Images of four clinical symptom subtypes of extrinsic adenomyosis. A) Pain, B) AUB, C) infertility, D) asymptomatic. The axial oblique plane was perpendicular to the endometrial cavity, resulting in a short-axis view.

In the study, a total of 405 patients diagnosed with extrinsic adenomyosis through MRI examination were enrolled based on the inclusion ang exclusion criteria. The patient’s flowchart was shown in Figure 1. The study workflow was shown in Figure 2. 405 patients presented with 496 clinical symptoms. Among them, totals of 173 (35%), 106 (21%), 84 (17%), and 133 (27%) patients were categorized into pain, AUB, infertility, and no symptom group, respectively. Finally, 121 of 284 patients (42.6%) and 52 of 121 (42.97%) patients with pain symptom were presented in the training and test sets, respectively, AUB in 75 of 284 (26.41%) and 31 of 121 (25.62%), infertility in 59 of 284 (20.77%) and 25 of 121 (20.66%), and no symptom in 95 of 284 and 38 of 121 (Table 1). The clinical characteristics of each symptom are presented in Table 2. There were no significant differences in age, weight, height, BMI, mean age at menarche, mean duration cycle, regular menstrual cycle, mean length of menstruations, endometriotic cyst, uterine myoma, history of miscarriage, previous uterine surgery between training and test sets. Figure 3 shows an example of extrinsic adenomyosis belonging to different symptom categories.

**Table 1.** Clinical symptoms in extrinsic adenomyosis in training and test cohorts.

	Training cohort (*n* = 284)	Test cohort (*n* = 121)	*P* value
Pain			0.65
Positive	121	52	
Negative	163	69	
Aub			0.12
Positive	75	31	
Negative	209	90	
Infertility			0.23
Positive	59	25	
Negative	225	96	
No symptom			0.34
Positive	95	38	
Negative	189	83	

**Table 2.** Clinical variables in training and test cohorts.

	Training cohort	Test cohort	P Value
Age, y	39.2(6.58)	40.7(6.54)	0.34
Weight, kg	56.5(7.40)	58.6(5.95)	0.23
Height, cm	159.1(4.65)	160.2(3.42)	0.21
BMI, kg/m^2^	22.2(2.46)	22.5(2.19)	0.51
Mean age at menarche, y	13.6(2.34)	14.5(2.44)	0.12
Mean duration cycle, d	28.8(3.86)	28.3(2.19)	0.43
Regular menstrual cycle (*n*%)	264(92.9)	100(82.7)	0.28
Mean length of menstruations, d	5.8(1.9)	6.4(1.8)	0.29
Endometriotic cyst (*n*%)	106(37.31)	51(42.1)	0.45
Uterine myoma (*n*%)	98(34.5)	45(37.1)	0.77
History of miscarriage (*n*%)	141(49.6)	55(45.5)	0.53
Previous uterine surgery (*n*%)	76(26.8)	45(37.1)	0.12

### Radiomics analysis and model construction

**Figure 4.**
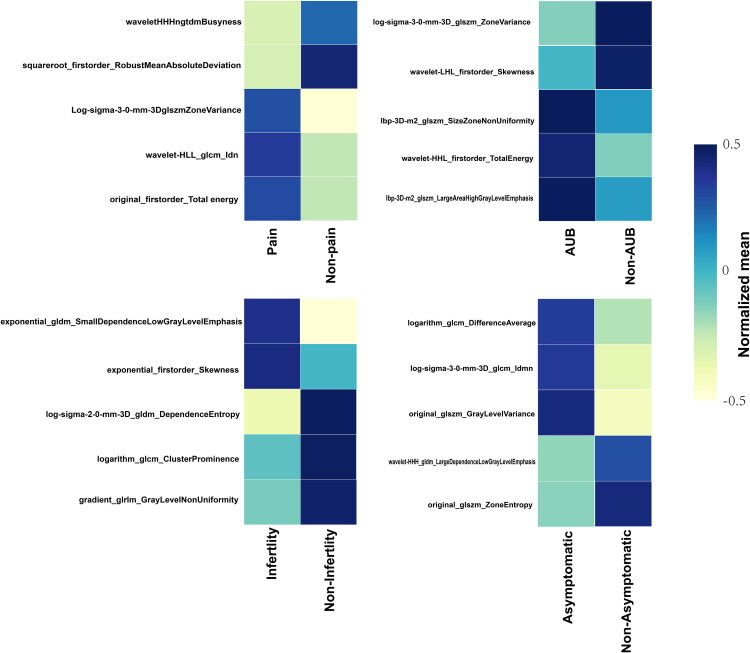
Normalized mean of radiomics features in four clinical symptom subtypes of extrinsic adenomyosis.

**Figure 5.**
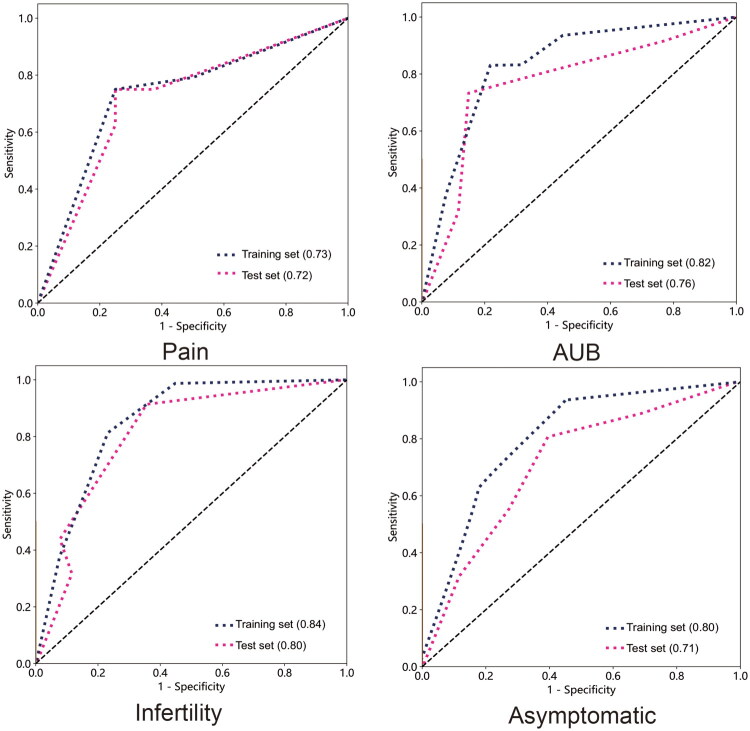
Receiver operating characteristic (ROC) curves in the training and the test sets for classifying (A) pain, (B)AUB, (C) infertility and (D) asymptomatic with use of radiomics-based SVM modeling. Areas under the ROC curves are reported in parentheses in the legend.

A total of 1474 radiomics features were extracted from T2WI sequence, consistency of the ROIs had the ICC below predefined 0.80 were exclude from next analysis, including 100 first-order,14 shape-based, 440 Gray-level co-occurrence matrix, 320 Gray-level size zone matrix, 320 Gray-level run length matrix, and 280 Gray-level dependence matrix features. We used a heatmap to determine the association in radiomics features in four different symptoms. There was no strong correlation (Spearman rank or point-biserial correlation coefficient, |0.5|) in these five selected features for dichotomizing symptom (Appendix Figures S1–S4). Based on four clinical symptom subtypes, Radom Forest (RF) algorithm was used to select the most discriminative radiomics feature. The top 5 most discriminative radiomics features based on RF algorithm, as ranked by the resulting AUC for each symptom task, were reported in Table 3, confirming that the RF selection process was able to detect important features. The top five selected features for distinguishing different symptoms of pain, AUB, infertility, and no symptom between the positive and negative groups. The following five radiomics features were finally selected: wavelet-HHH ngtdm Busyness, squareroot_firstorder_RobustMeanAbsoluteDeviation, Log-sigma-3-0-mm-3D glszm ZoneVariance, wavelet-HLL_glcm_Idn, original_firstorder_Total energy in pain; log-sigma-3-0-mm-3D_glszm_ZoneVariance, wavelet-LHL_firstorder_Skewness, lbp 3Dm2_glszm_Size Zone Non Uniformity, wavelet-HHL_firstorder_TotalEnergy, lbp-3Dm2_glszm_Large Area High Gray Level Emphasis in AUB; exponential_gldm_Small Dependence Low Gray Level Emphasis, exponential_firstorder_Skewness, log-sigma-2-0-mm-3D_gldm_DependenceEntropy, logarithm_glcm_Cluster Prominence, gradient_glrlm_Gray Level Non Uniformity in infertility, logarithm_glcm_Difference Average, log-sigma-3-0-mm-3D_glcm_Idmn, original_glszm_Gray Level Variance, wavelet-HHH_gldm_Large Dependence Low Gray Level Emphasis, original_glszm_Zone Entropy in no symptom. The Normalized mean of five selected features for distinguishing different symptoms were list in Figure 4. Simultaneously, incorporating the key features of ROI *via* SVM for pain symptom achieved an AUC of 0.73, 0.72, AUB symptom achieved an AUC of 0.82, 0.76, infertility symptom achieved an AUC of 0.84, 0.80, no symptom achieved an AUC of 0.80, 0.71 in the training and test sets. The detailed evaluation indicators for model performance including sensitivity and specificity are summarized in Table 4. ROC curves were presented in Figure 5. Rad-core was calculated according to the formula (Appendix E1).

**Table 3.** Description of selected radiomics features in random Forest and their importance for symptom classification.

Symptoms and radiomics feature	AUC (Training)	AUC (Test)	Feature importance	*P* value
***Pain***				
wavelet-HHH ngtdm Busyness	0.69(0.54, 0.78)	0.67(0.64, 0.86)	0.043	<0.01
squareroot_firstorder_RobustMeanAbsoluteDeviation	0.68(0.57, 0.86)	0.69(0.65, 0.77)	0.020	<0.01
Log-sigma-3-0-mm-3D glszm ZoneVariance	0.68(0.48, 0.78)	0.66(0.52, 0.73)	0.017	<0.01
wavelet-HLL_glcm_Idn	0.67(0.54, 0.73)	0.63(0.39, 0.81)	0.016	<0.01
original_firstorder_Total energy	0.64(0.53, 0.75)	0.62(0.52, 0.82)	0.015	<0.01
***AUB***				
log-sigma-3-0-mm-3D_glszm_ZoneVariance	0.72(0.46, 0.78)	0.70(0.62, 0.74)	0.039	<0.01
wavelet-LHL_firstorder_Skewness	0.71(0.63, 0.81)	0.70(0.61, 0.83)	0.037	<0.01
lbp-3D-m2_glszm_SizeZoneNonUniformity	0.70(0.52, 0.78)	0.66(0.63, 0.86)	0.031	<0.01
wavelet-HHL_firstorder_TotalEnergy	0.68(0.54, 0.77)	0.66(0.56, 0.76)	0.029	<0.01
lbp-3D-m2_glszm_LargeAreaHighGrayLevelEmphasis	0.65(0.47, 0.82)	0.64(0.51, 0.73)	0.027	<0.01
***Infertility***				
exponential_gldm_SmallDependenceLowGrayLevelEmphasis	0.75(0.63, 0.83)	0.76(0.54, 0.78)	0.031	<0.01
exponential_firstorder_Skewness	0.76(0.54, 0.78)	0.75(0.72, 0.87)	0.028	<0.01
log-sigma-2-0-mm-3D_gldm_DependenceEntropy	0.75(0.62, 0.86)	0.73(0.49, 0.76)	0.027	<0.01
logarithm_glcm_ClusterProminence	0.74(0.58, 0.81)	0.72(0.46, 0.77)	0.020	<0.01
gradient_glrlm_GrayLevelNonUniformity	0.71(0.63, 0.85)	0.68(0.66, 0.84)	0.018	<0.01
***No symptom***				
logarithm_glcm_DifferenceAverage	0.74(0.45, 0.76)	0.72(0.60, 0.82)	0.034	<0.01
log-sigma-3-0-mm-3D_glcm_Idmn	0.74(0.63, 0.87)	0.74(0.64, 0.79)	0.030	<0.01
original_glszm_GrayLevelVariance	0.70(0.60, 0.81)	0.69(0.41, 0.64)	0.029	<0.01
wavelet-HHH_gldm_LargeDependenceLowGrayLevelEmphasis	0.68(0.46, 0.76)	0.67(0.54, 0.84)	0.026	<0.01
original_glszm_ZoneEntropy	0.67(0.57, 0.86)	0.65(0.53, 0.76)	0.019	<0.01

**Table 4.** Diagnostic performance of support vector machine for symptom stratification.

Symptom and data set	AUC	Sensitivity (%)	Specificity (%)	Accuracy (%)	PPV (%)	NPV (%)	*P* value
Pain							0.02
Training	0.73(0.68, 0.89)	87	74	80	80	82	
Test	0.72(0.67, 0.84)	82	90	68	90	68	
AUB							0.03
Training	0.82(0.68, 0.86)	84	79	86	84	88	
Test	0.76(0.64, 0.82)	86	75	80	82	80	
Infertility							0.03
Training	0.84(0.62, 0.81)	84	88	86	94	88	
Test	0.80(0.63, 0.85)	86	84	79	73	83	
No symptom							<0.01
Training	0.80(0.64, 0.82)	84	84	83	82	80	
Test	0.71(0.61, 0.87)	84	82	76	89	78	

### Clinical characteristics analysis

We summarized the clinical characteristics in different symptom (pain, AUB, infertility, and no symptom). Univariable analyses revealed that statistically significant clinical characteristics were related to each symptom, as shown in Appendix Tables S1–S4. Multivariable logistic regression analysis identified that age (OR1.32, 95%CI 1.23–1.65), DIE (OR2.05, 95%CI 1.64–2.34), CA125 (OR1.43, 95%CI 1.15–2.79) were independent clinical risk predictors in pain group. In AUB group, BMI (OR 2.14 95%CI 1.67–2.34), regular menstrual cycle (OR1.78, 95%CI 1.45–1.86), history of miscarriage (OR1.66, 95%CI 1.23–1.87) were independent clinical risk predictors. In infertility group, age (OR 2.54, 95%CI 1.56–2.62), BMI (OR 1.42,95%CI 1.23–1.76), regular menstrual cycle (OR 1.23, 95%CI 1.15–1.65), endometriotic cyst (OR 1.87, 95%CI 1.45–2.05) were independent clinical risk predictors. In no symptom group, age (OR 0.64, 95%CI 0.56–0.82), gravidity (OR 0.53,95%CI 0.45–0.78), endometriotic cyst (OR 0.47,95%CI 0.44–0.82), junctional zone size (OR 0.49,95%CI 0.25–0.64) were independent clinical protective predictors. These clinical characteristics were found to be significant difference in multivariate logistic regression analysis of each symptom (Table 5).

**Table 5.** Multivariable analysis of clinical characteristics for symptom stratification.

Clinical symptom	OR	95%CI	*P* value
Pain			
Age, y	1.32	1.23–1.65	0.018
DIE	2.05	1.64–2.34	0.025
CA125	1.43	1.15–2.79	0.016
AUB			
BMI, kg/m^2^	2.14	1.67–2.34	0.035
Regular menstrual cycle	1.78	1.45–1.86	0.023
History of miscarriage	1.66	1.23–1.87	0.034
Infertility			
Age, y	2.54	1.56–2.62	0.015
BMI, kg/m^2^	1.42	1.23–1.76	0.013
Regular menstrual cycle	1.23	1.15–1.65	0.014
Endometriotic cyst	1.87	1.45–2.05	0.013
No symptom			
Age, y	0.64	0.56–0.82	0.031
Gravidity	0.53	0.45–0.78	0.024
Endometriotic cyst	0.47	0.44–0.82	0.021
Junctional zone size (mm)	0.49	0.25–0.64	0.017

### Construction of clinical–radiomics combined model

**Figure 6.**
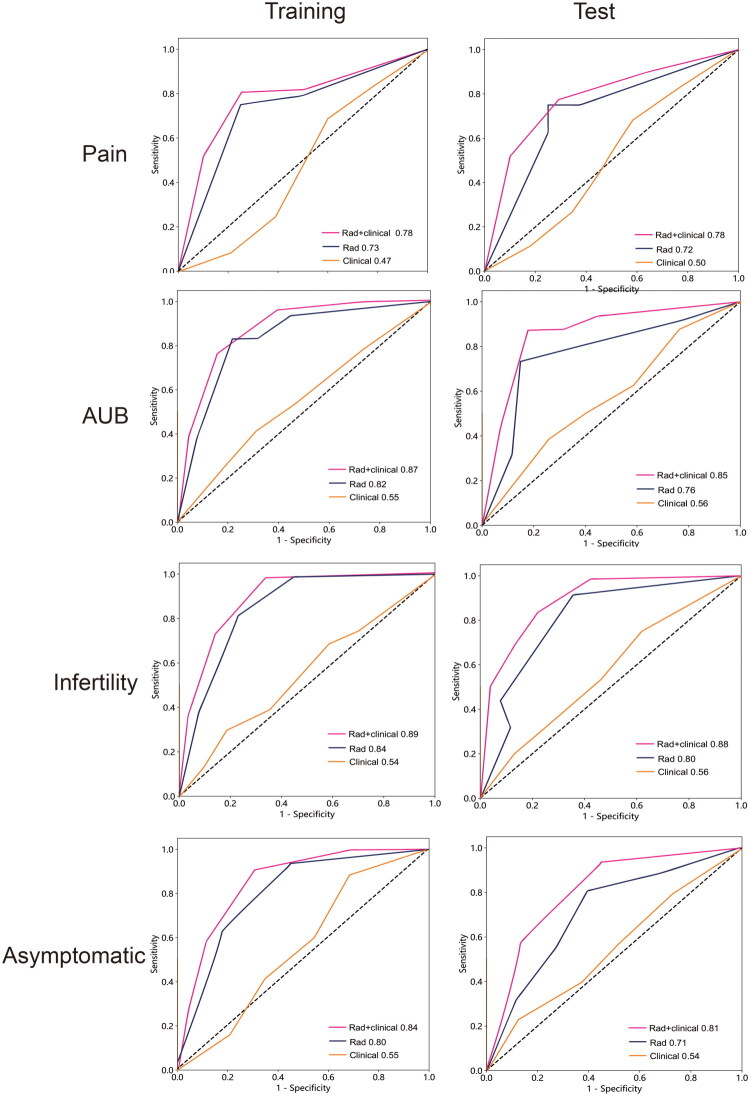
Receiver operating characteristic (ROC) curves in the training and the test sets for classifying (A) pain, (B) AUB, (C) infertility and (D) asymptomatic with use of radiomics–clinical modeling.

**Figure 7.**
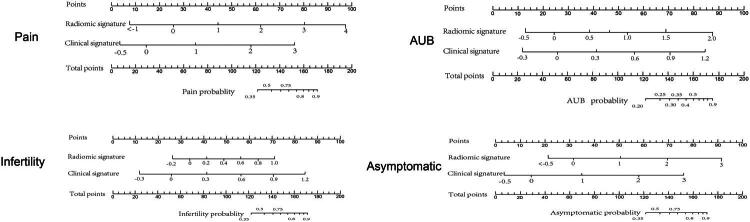
A nomogram for predicted symptom combined with clinical characteristics and Rad-model.

Afterward, based on selected independent radiomic and clinical features, to build radiomic/clinical and radiomic–clinical models. The performance of each model evaluated by ROC are shown in Table 6 and Figure 6. Adding clinical characteristics to rad-model can enhance efficiency. The AUCs of radiomic–clinical of pain, AUB, infertility, and no symptom group were 0.78, 0.87, 0.89, 0.84 in training cohort and 0.78, 0.85, 0.88, 0.81 in test cohort. The clinical–radiomics combined model showed significantly improved performance for predicting symptom compared with the clinical/radiomic model both in the training cohort (*p* < 0.05) and in the validation cohort (*p* < 0.05). A nomogram was constructed based on Rad-score and clinical score in Figure 7. Clinical score calculation formula was shown in Appendix E2. The calibration chart demonstrated a strong correlation between the nomogram’s predicted outcomes and the actual data for patients across both the training and validation sets. Figure S5 displayed the decision curve analysis for the radiomics nomogram. This curve indicates that for a patient utilizing the radiomics nomogram to forecast symptoms, there is a greater benefit than adopting a strategy of either no treatment or treating all patients. Within this probability threshold, the net benefit, as determined by the radiomics nomogram, was found to be similar, with some areas of overlap.

**Table 6.** Performance of symptom prediction models in training and test cohorts.

		Training cohort			Test cohort		
Symptom	Models	Sensitivity (%)	Specificity (%)	AUC	Sensitivity (%)	Specificity (%)	AUC
Pain	Clinical Model	87	76	0.47(0.43, 0.56)	65	86	0.50(0.47, 0.63)
	Radiomic Model	67	78	0.73(0.54, 0.78)	65	78	0.72(0.64, 0.74)
	Clinical + Radiomic Model	74	78	0.78(0.64, 0.80)	78	85	0.78(0.62, 0.82)
AUB	Clinical Model	65	76	0.55(0.49, 0.64)	67	75	0.56(0.52, 0.64)
	Radiomic Model	62	64	0.82(0.72, 0.86)	62	71	0.76(0.55, 0.78)
	Clinical + Radiomic Model	65	72	0.87(0.74, 0.87)	65	72	0.85(0.64,0.86)
Infertility	Clinical Model	62	68	0.54(0.48, 0.64)	72	72	0.56(0.53, 0.65)
	Radiomic Model	67	72	0.84(0.64, 0.86)	67	85	0.80(0.58, 0.82)
	Clinical + Radiomic Model	71	76	0.89(0.64, 0.89)	66	72	0.88(0.67, 0.88)
No symptom	Clinical Model	63	72	0.55(0.46, 0.61)	64	67	0.54 (0.51, 0.78)
	Radiomic Model	61	72	0.80(0.65, 0.86)	65	73	0.71(0.67, 0.85)
	Clinical + Radiomic Model	63	68	0.84(0.75, 0.90)	63	76	0.81(0.75, 0.91)

## Discussion

Detection of adenomyosis symptom using a noninvasive and widely available technique is very important, as it may help develop more personalized treatment options in wide populations [12]. The may finding of our study was the radiomics signature related with the symptom of extrinsic adenomyosis. In addition, selected radiomics feature for discriminating symptom heterogeneity beyond conventional clinical characteristics. In this study, we established radiomics combined with clinical characteristics on MRI for stratification of extrinsic adenomyosis according to the clinical symptomatology.

Radiomics was an innovative technology to get mineable high-dimensional image data that related to disease pathophysiology. Studies suggested that radiomic features are strongly correlated with tissue heterogeneity to be directly linked with histological features, molecular mechanism, immune microenvironment. This information was aid in treatment planning, suggested to predict therapeutic efficacy, clinical outcome. Therefore, radiomics built the bridge between the routine medical images and personal clinical-decision support. Manual ROI segmentation was time consuming and limited reproducibility. Therefore, deep learning was utilized to develop more accurate and efficient automated segmentation algorithms to overcome the limitations of manual segmentation. Previous studies have revealed texture-feature GLCM inverse difference was founded to be with poor outcome in lung cancer [13]. This feature was positively related with CAIX expression, a central enzyme upregulated in tumor hypoxia of tumoral microenvironment. Selected radiomics can be used in achieving machine learning model algorithms predicting the overall survival and immune response in lung cancer [14]. Fan’s study conducted a radiomic model in breast cancer to predict treatment response and pathological outcomes enable clinicians enhance the efficiency of treatment, reduce the medical cost [15]. An axillary lymph node (ALN)-tumor radiomic model showed a higher prediction of preoperative individual’s ALN status by a non-invasive method [7]. The key radiomics features worked as image biomarkers were correlated with immune cells, methylated sites, which involved in potential biological progress. According to the results of the current studies, MRI-based radiomics demonstrated a potential promising technique for improving adenomyosis diagnosis [16]. Multi-machine learning model could be predicted treat outcome in magnetic resonance-guided focused ultrasound ablation and uterine artery embolization therapy [8,17,18]. To date, there have been no studies based on imaging histology targeting symptom stratification of adenomyosis.

Adenomyosis diagnosed by MRI demonstrated a higher accuracy sensitivity and specificity. MRI showed a better identification of morphologic features of this disease. MRI can present the lesion distribution of different subtypes of adenomyosis [19]. Developing a classification of adenomyosis linked with symptoms related to pathology will beneficial to the comprehension of the disease. The International Federation of Gynecology and Obstetrics (FIGO) restarted work on a classification system for adenomyosis in 2015, but it is still in draft form [20]. The thickness of junctional zone over than 12 mm was consider as constructive diagnostic evidence. MRI parameters JZ thickness, myometrial involvement, focal or diffuse could be include in heterogeneous subtypes [21]. Kishi’s description categorized extrinsic adenomyosis arose in the outer shell of the uterus disrupting the serosa but not affecting the inner components, exhibit more easily with pain symptom (dysmenorrhea, non-cyclic pelvic pain and dyspareunia). Our clinical experience has indicated that there are still multiple heterogeneous clinical profiles in extrinsic adenomyosis, consistent with previous studies. Habiba etl have attempted to evaluate the symptoms correlated with ultrasound features [22]. But they were unable to quantify the lesions and related symptoms. Compared with ultrasound, MRI images showed superior tissue differentiation and detailed anatomical structure. In our study, the radiomic model dichotomized pain, AUB, infertility, and asymptomatic led to the areas under the curves (AUCs) 0.73, 0.82, 0.84, and 0.80 in the training cohort, respectively, 0.72, 0.80, 0.82, and 0.80 in the test cohort. The combined clinical–radiomic nomogram showed strong discrimination of pain, AUB, infertility, and asymptomatic with the AUCs of 0.78, 0.84, 0.87, 0.89 in the training and 0.78, 0.81, 0.85, 0.88 in the test cohort. The GLCM features can be interpreted as a measure of skewness, reflecting the presence of large variation and high grayscale levels. Additionally, GLCM features can characterize the contrast, intricacy, and heterogeneity of local strength modes, potentially indicating the proliferative heterogeneity of adenomyosis lesion. MRI images exhibit high reproducibility, and the standardized images obtained facilitate the construction of stable radiomics models [23]. It will beneficial for follow-up diagnosis and treatment of adenomyosis

The heterogeneity of clinical symptoms in adenomyosis influences the selection of treatment protocols and the achievement of therapeutic goals [24–26]. The majority of medical interventions aim to regulate estrogen and progesterone levels, thereby inhibiting the overgrowth of the endometrial lining and reducing the production of inflammatory cytokines and prostaglandins [27]. Our clinical experience indicated that the therapeutic outcomes of medication can be inconsistent when treating patients with subtypes of adenomyosis [4]. Advancements in GnRH-a therapy are rapid, but they can lead to side effect symptoms like hot flashes, potentially impacting patient compliance and the feasibility of long-term treatment [28]. Progesterone therapy proved effective in treating patients with extrinsic adenomyosis [29]. Khan and colleagues assessed the presence of estrogen and progesterone receptors in the tissue samples from patients with intrinsic and extrinsic adenomyosis[19]. They found that progesterone receptor cells were significantly lower than the estrogen receptor cells in intrinsic adenomyosis (*p* < 0.05), but the expression levels of progesterone and estrogen receptors were found to be equivalent in the extrinsic subtype. On the other hand, extrinsic adenomyosis may be exhibit heterogeneity regarding the quantity of progesterone receptors. In this contest, some investigators forwarded to explain the pathogenesis of variant subtype adenomyosis [30]. An increased risk of miscarriage in early pregnancy is associated with adenomyosis, supporting the theory of suboptimal implantation. Adenomyosis are more likely associated with placental abruption, preterm delivery, postpartum hemorrhage, small for gestational age fetus in late pregnancy, suggesting the possible implantation and placentation abnormalities [31]. Among radiomic features, factors were important components for predicting histologic pathogenesis of adenomyosis. Therefore, MRI can serve a comprehensive role throughout the diagnosis, treatment decision-making, and medical care processes, enhancing patient symptoms while minimizing unnecessary medical expenses.

Our study still had several limitations. First, our sample was relatively small enrolled from a single center. Second, our study was a retrospective study, and there may be bias in the selection of cases. Third, the ROI drawn by clinicians was time-consuming and labor-intensive. In the future, large sample, multi-center cohorts should be conduct. A method for automatically segmenting ROI is need to develop while alleviating the pressure of radiology department. The biologic meaning of radiomic features also must be better understood to bridge radiomics and the histologic characteristics of adenomyosis lesion.

## Conclusion

In conclusion, this study presented radiomics features of adenomyosis lesion from MRI that related with symptom of extrinsic adenomyosis. Our clinical–radiomic models provided consistent clinically acceptable performance for differentiating symptom heterogeneity. Our classifier had a better performance alleviate the workload of imaging physicians and may serve as a promising noninvasive imaging marker in adenomyosis stratification in future clinical practice. Future studies should assess the relationship between radiomics features and pathogenesis and explore the molecular mechanism of different symptom of extrinsic adenomyosis.

## Supplementary Material

Supplemental Material

## Data Availability

The datasets generated during and/or analyzed during the current study are not publicly available due to patient permission was not sought for the sharing of data at the time of recruitment but are available from the corresponding author on reasonable request.

## References

[1] Chapron C, Vannuccini S, Santulli P, et al. Diagnosing adenomyosis: an integrated clinical and imaging approach. Hum Reprod Update. 2020;26(3):392–411. doi: 10.1093/humupd/dmz049. 32097456

[2] Bourdon M, Oliveira J, Marcellin L, et al. Adenomyosis of the inner and outer myometrium are associated with different clinical profiles. Hum Reprod. 2021;36(2):349–357. doi: 10.1093/humrep/deaa307. 33491057

[3] Zhang M, Bazot M, Tsatoumas M, et al. MRI of Adenomyosis: where Are We Today? Can Assoc Radiol J. 2023;74(1):58–68. doi: 10.1177/08465371221114197. 35856446

[4] Han X, Gao X, Wang F, et al. Heterogeneity of clinical symptoms and therapeutic strategies for different subtypes of adenomyosis: an initial single-center study in China. Int J Gynaecol Obstet. 2023;161(3):775–783. doi: 10.1002/ijgo.14650. 36605017

[5] Borghese G, Doglioli M, Orsini B, et al. Progression of adenomyosis: rate and associated factors. Int J Gynaecol Obstet. 2024;167(1):214–222. doi: 10.1002/ijgo.15572. 38738458

[6] Mayerhoefer ME, Materka A, Langs G, et al. Introduction to radiomics. J Nucl Med. 2020;61(4):488–495. doi: 10.2967/jnumed.118.222893. 32060219 PMC9374044

[7] Yu Y, He Z, Ouyang J, et al. Magnetic resonance imaging radiomics predicts preoperative axillary lymph node metastasis to support surgical decisions and is associated with tumor microenvironment in invasive breast cancer: a machine learning, multicenter study. EBioMedicine. 2021;69:103460. doi: 10.1016/j.ebiom.2021.103460. 34233259 PMC8261009

[8] Li Z, Zhang J, Song Y, et al. Utilization of radiomics to predict long-term outcome of magnetic resonance-guided focused ultrasound ablation therapy in adenomyosis. Eur Radiol. 2021;31(1):392–402. doi: 10.1007/s00330-020-07076-1. 32725335

[9] Kishi Y, Suginami H, Kuramori R, et al. Four subtypes of adenomyosis assessed by magnetic resonance imaging and their specification. Am J Obstet Gynecol. 2012;207(2):114.e111–114.e117. doi: 10.1016/j.ajog.2012.06.027. 22840719

[10] Guiot J, Vaidyanathan A, Deprez L, et al. A review in radiomics: making personalized medicine a reality via routine imaging. Med Res Rev. 2022;42(1):426–440. doi: 10.1002/med.21846. 34309893

[11] Mishra P, Pandey CM, Singh U, et al. Descriptive statistics and normality tests for statistical data. Ann Card Anaesth. 2019;22(1):67–72. doi: 10.4103/aca.ACA_157_18. 30648682 PMC6350423

[12] Gordts S, Grimbizis G, Campo R. Symptoms and classification of uterine adenomyosis, including the place of hysteroscopy in diagnosis. Fertil Steril. 2018;109(3):380–388.e1. doi: 10.1016/j.fertnstert.2018.01.006. 29566850

[13] Chen M, Copley SJ, Viola P, et al. Radiomics and artificial intelligence for precision medicine in lung cancer treatment. Semin Cancer Biol. 2023;93:97–113. doi: 10.1016/j.semcancer.2023.05.004. 37211292

[14] Luan J, Zhang D, Liu B, et al. Immune-related lncRNAs signature and radiomics signature predict the prognosis and immune microenvironment of glioblastoma multiforme. J Transl Med. 2024;22(1):107. doi: 10.1186/s12967-023-04823-y. 38279111 PMC10821572

[15] Fan M, Wang K, Pan D, et al. Radiomic analysis reveals diverse prognostic and molecular insights into the response of breast cancer to neoadjuvant chemotherapy: a multicohort study. J Transl Med. 2024;22(1):637. doi: 10.1186/s12967-024-05487-y. 38978099 PMC11232151

[16] Burla L, Sartoretti E, Mannil M, et al. MRI-based radiomics as a promising noninvasive diagnostic technique for adenomyosis. J Clin Med. 2024;13(8):2344. doi: 10.3390/jcm13082344. 38673617 PMC11051471

[17] Ying J, Jing X, Gao F, et al. Prediction of ablation rate for high-intensity focused ultrasound therapy of adenomyosis in MR images based on multi-model fusion. J Imaging Inform Med. 2024;37(4):1579–1590. doi: 10.1007/s10278-024-01063-4. 38441701 PMC11300765

[18] Jin W, Wang S, Wang T, et al. Multi-machine learning model based on habitat subregions for outcome prediction in adenomyosis treated by uterine artery embolization. Acad Radiol. 2024;31(12):4985–4995. doi: 10.1016/j.acra.2024.05.037. 38845295

[19] Khan KN, Fujishita A, Koshiba A, et al. Biological differences between intrinsic and extrinsic adenomyosis with coexisting deep infiltrating endometriosis. Reprod Biomed Online. 2019;39(2):343–353. doi: 10.1016/j.rbmo.2019.03.210. 31160242

[20] Habiba M, Gordts S, Bazot M, et al. Exploring the challenges for a new classification of adenomyosis. Reprod Biomed Online. 2020;40(4):569–581. doi: 10.1016/j.rbmo.2020.01.017. 32173239

[21] Novellas S, Chassang M, Delotte J, et al. MRI characteristics of the uterine junctional zone: from normal to the diagnosis of adenomyosis. AJR Am J Roentgenol. 2011;196(5):1206–1213. doi: 10.2214/AJR.10.4877. 21512093

[22] Habiba M, Benagiano G. The incidence and clinical significance of adenomyosis. In: Habiba M, Benagiano G, editors. Uterine adenomyosis. Cham: Springer International Publishing; 2016. p. 9–43.

[23] Levy G, Dehaene A, Laurent N, et al. An update on adenomyosis. Diagn Interv Imaging. 2013;94(1):3–25. doi: 10.1016/j.diii.2012.10.012. 23246186

[24] Zhang B, Shi J, Gu Z, et al. The role of different LNG-IUS therapies in the management of adenomyosis: a systematic review and meta-analysis. Reprod Biol Endocrinol. 2025;23(1):23. doi: 10.1186/s12958-025-01349-4. 39948612 PMC11823221

[25] Moawad G, Kheil MH, Ayoubi JM, et al. Adenomyosis and infertility. J Assist Reprod Genet. 2022;39(5):1027–1031. doi: 10.1007/s10815-022-02476-2. 35347501 PMC9107544

[26] Ji M, Yuan M, Jiao X, et al. A cohort study of the efficacy of the dienogest and the gonadotropin-releasing hormone agonist in women with adenomyosis and dysmenorrhea. Gynecol Endocrinol. 2022;38(2):164–169. doi: 10.1080/09513590.2021.2000961. 34749585

[27] Bourdon M, Sorel M, Maignien C, et al. Progesterone levels do not differ between patients with or without endometriosis/adenomyosis both in those who conceive after hormone replacement therapy–frozen embryo transfer cycles and those who do not. Hum Reprod. 2024;39(8):1692–1700. doi: 10.1093/humrep/deae114. 38850031

[28] Che X, Wang J, Sun W, et al. Effect of mifepristone vs placebo for treatment of adenomyosis with pain symptoms: a randomized clinical trial. JAMA Netw Open. 2023;6(6):e2317860. doi: 10.1001/jamanetworkopen.2023.17860. 37307001 PMC10261993

[29] Vannuccini S, Luisi S, Tosti C, et al. Role of medical therapy in the management of uterine adenomyosis. Fertil Steril. 2018;109(3):398–405. doi: 10.1016/j.fertnstert.2018.01.013. 29566852

[30] Bulun SE, Yildiz S, Adli M, et al. Adenomyosis pathogenesis: insights from next-generation sequencing. Hum Reprod Update. 2021;27(6):1086–1097. doi: 10.1093/humupd/dmab017. 34131719 PMC8543024

[31] Horton J, Sterrenburg M, Lane S, et al. Reproductive, obstetric, and perinatal outcomes of women with adenomyosis and endometriosis: a systematic review and meta-analysis. Hum Reprod Update. 2019;25(5):592–632. doi: 10.1093/humupd/dmz012. 31318420

