# Supplementary material for: Development and validation of MRI-based radiomics model for clinical symptom stratification of extrinsic adenomyosis
Source: Ann Med. 2025 Jul 25;57(1):2534521. doi: 10.1080/07853890.2025.2534521 (PMC12302386; doi:10.1080/07853890.2025.2534521)

#### Supplementary materials

Appendix E1 radscore calculation formula

Pain label = 0.484-0.02733*waveletHHHngtdmBusyness

-0.004975*squareroot_firstorder_RobustMeanAbsoluteDeviation

+0.045413 * Log-sigma-3-0-mm-3DglszmZoneVariance

+0.029567*wavelet-HLL_glcm_Idn

+0.031518 *original_firstorder_Total energy

AUB label= 0.7853-0.056782 * log-sigma-3-0-mm-3D_glszm_ZoneVariance

-0.073276 * wavelet-LHL_firstorder_Skewness

+0.039656 * lbp-3D-m2_glszm_SizeZoneNonUniformity

+0.056* wavelet-HHL_firstorder_TotalEnergy

+ 0.048 lbp-3D-m2_glszm_LargeAreaHighGrayLevelEmphasis

Infertility label= 0.6880

+0.112exponential_gldm_SmallDependenceLowGrayLevelEmphasis

+0.172 exponential_firstorder_Skewness

-0.045log-sigma-2-0-mm-3D_gldm_DependenceEntropy

- 0.267logarithm_glcm_ClusterProminence

-0.218 gradient_glrlm_GrayLevelNonUniformity

Asymptomatic label= 0.6555+0.0273 logarithm_glcm_DifferenceAverage

+0.0295 log-sigma-3-0-mm-3D_glcm_Idmn

+0.0315 original_glszm_GrayLevelVariance

-0.0049 wavelet-HHH_gldm_LargeDependenceLowGrayLevelEmphasis

-0.0567 original_glszm_ZoneEntropy

| Table S1 Univariate Analyses of clinical characteristics related with pain | | | | |  |
| --- | --- | --- | --- | --- | --- |
| Clinical Variable | positive （173） | negative（232） | OR | 95%CI | P-value |
| age, y | 38.52±7.17 | 34.73±4.76 | 1.82 | 1.34-3.06 | *0.03* |
| height, cm | 160.48±6.52 | 160.85±4.23 | 1.15 | 0.73-2.31 | 0.95 |
| weight, kg | 62.09±5.53 | 63.34±4.34 | 1.14 | 0.45-2.62 | 0.92 |
| BMI, kg/m2 | 22.12±2.78 | 22.61±1.89 | 1.87 | 0.76-1.19 | 0.87 |
| Gravidity | 2.04±0.65 | 1.98±0.33 | 1.14 | 0.81-3.43 | 0.64 |
| Parity | 1.45±0.67 | 1.75±0.25 | 0.94 | 0.76-3.52 | 0.63 |
| mean age at menarche, y | 14.07±1.74 | 14.74±1.81 | 1.35 | 1.67-5.22 | 0.67 |
| mean duration cycle, d | 31.52±5.54 | 29.85±5.87 | 1.32 | 0.83-1.67 | 0.75 |
| mean length of menstruations, d | 6.37±1.53 | 6.11±1.33 | 0.77 | 0.78-3.11 | 0.73 |
| regular menstrual cycle (n, %) | 155, 89.60 | 207, 89.22 | 0.92 | 0.86-2.17 | 0.64 |
| Endometriotic cyst (n, %) | 93, 53.76 | 104, 44.83 | 1.73 | 1.64-2.96 | 0.03 |
| Deep infiltrating endometriosis (n, %) | 82, 47.39 | 72, 32.33 | 2.86 | 1.64-4.35 | *<0.01* |
| Uterine myoma (n, %) | 83, 47.98 | 106, 45.69 | 1.34 | 0.71-1.58 | 0.62 |
| history of miscarriage (n, %) | 40, 23,12 | 58, 25 | 1.53 | 0.71-3.64 | 0.81 |
| previous uterine surgery (n, %)) | 75, 43.35 | 86, 37.07 | 1.29 | 0.48-2.68 | 0.78 |
| CA125 | 98.24±10.99 | 56.78±8.84 | 1.79 | 1.43-2.93 | *0.02* |
| Junctional zone size (mm) | 6.71±2.23 | 5.9±3,53 | 1.45 | 0.76-3,41 | 0.62 |
| Junctional zone/myometrium ratio | 0.38±0.14 | 0.37±0.12 | 1.46 | 0.67-2.41 | 0.75 |
| Mean myometrium (mm) | 30.57±5.45 | 23.12±5.78 | 1.32 | 0.83-5.45 | 0.62 |

| Table S2 Univariate Analyses of clinical characteristics related with AUB | | | | | |
| --- | --- | --- | --- | --- | --- |
| Clinical Variable | Positive (107) | Negative (298) | OR | 95%CI | P-value |
| age, y | 37.52±3.74 | 38.73±4.18 | 1.34 | 0.65-3.42 | 0.28 |
| height, cm | 160.75±6.23 | 159.23±6.86 | 1.45 | 0.73-4.21 | 0.27 |
| weight, kg | 60.04±4.84 | 59.38±4.14 | 1.37 | 0.68-3.78 | 0.45 |
| BMI, kg/m2 | 24.69±3.75 | 21.81±3.08 | 2.12 | 1.53-3.61 | *<0.01* |
| Gravidity | 2.13±0.57 | 2.08±0.63 | 1.42 | 0.64-3.42 | 0.63 |
| Parity | 1.34±0.43 | 1.52±0.23 | 1.16 | 0.75-2.32 | 0.72 |
| mean age at menarche, y | 14.46±1.01 | 14.78±0.94 | 1.23 | 0.67-2.35 | 0.53 |
| mean duration cycle, d | 38.52±6.03 | 30.64±7.34 | 1.34 | 0.75-3.46 | 0.03 |
| mean length of menstruations, d | 7.19±2.34 | 6.62±2.71 | 1.26 | 0.56-2.63 | 0.76 |
| regular menstrual cycle (n, %) | 86, 80.37 | 276, 92.62 | 0.64 | 0.42-0.73 | *0.02* |
| Endometriotic cyst (n, %) | 53, 49.53 | 144, 48.32 | 1.32 | 0.72-4.35 | 0.64 |
| Deep infiltrating endometriosis | 37, 34.26 | 117, 39.26 | 1.43 | 0.63-2.83 | 0.54 |
| Uterine myoma (n, %) | 45, 42.06 | 144, 48.32 | 1.28 | 0.78-3.57 | 0.52 |
| history of miscarriage (n, %) | 42 39.26 | 56, 18.79 | 1.76 | 1.32-2.65 | *0.02* |
| previous uterine surgery (n, %)) | 75, 43.35 | 85, 36.64 | 1.65 | 0.53-3.21 | 0.64 |
| CA125 | 64.65±23.25 | 61.33±22.06 | 1.14 | 0.71-2.39 | 0.63 |
| Junctional zone size (mm) | 8.43±1.81 | 4.78±2.12 | 1.67 | 0.68-2.45 | 0.54 |
| Mean myometrium (mm) | 33.72±9.83 | 22.25±7.73 | 1.89 | 0.65-2.32 | 0.47 |
| Junctional zone/myometrium ratio | 0.48±0.27 | 0.21±0.12 | 1.21 | 0.72-2.28 | 0.66 |

| Table S3 Univariate Analyses of clinical characteristics related with infertility | | | | | |
| --- | --- | --- | --- | --- | --- |
| Clinical characteristics | positive (74) | negative (331) | OR | 95%CI | P-value |
| age, y | 38.31±6.27 | 34.7±3.74 | 1.51 | 1.34-2.54 | 0.03 |
| height, cm | 160.24±5.26 | 160.75±3.86 | 1.72 | 0.78-2.17 | 0.33 |
| weight, kg | 57.08±8.18 | 56.38±8.14 | 1.46 | 0.65-2.12 | 0.57 |
| BMI, kg/m2 | 23.25±3.14 | 21.82±2.08 | 1.95 | 1.78-3.56 | 0.02 |
| Gravidity | 0.88±0.37 | 2.09±0.32 | 0.98 | 0.67-1.46 | 0.41 |
| Parity | 0.57±0.46 | 1.73±0.55 | 1.04 | 0.65-1.56 | 0.47 |
| mean age at menarche, y | 14.69±1.02 | 14.78±0.94 | 1.03 | 0.73-2.43 | 0.19 |
| mean duration cycle, d | 29.67±7.34 | 28.65±4.92 | 1.97 | 0.75-3.46 | 0.33 |
| mean length of menstruations, d | 7.35±5.82 | 6.27±1.72 | 1.21 | 0.74-2.14 | 0.19 |
| regular menstrual cycle (n, %) | 49, 66.22 | 313, 86.70 | 0.78 | 0.45-0.86 | 0.01 |
| Endometriotic cyst (n, %) | 36, 48.65 | 161, 44.60 | 1.49 | 1.25-2.16 | 0.02 |
| Deep infiltrating endometriosis (n, %) | 25, 33.78 | 129, 35.73 | 0.93 | 0.74-1.62 | 0.83 |
| Uterine myoma (n, %) | 33, 44.59 | 156, 43.21 | 0.98 | 0.46-1.35 | 0.92 |
| history of miscarriage (n, %) | 17, 18.92 | 81, 22.44 | 1.48 | 0.68-1.89 | 0.47 |
| previous uterine surgery (n, %)) | 30, 40.54 | 131, 36.29 | 1.72 | 0.72-1.43 | 0.52 |
| CA125 | 79.8±17.68 | 61.00±12.80 | 1.59 | 1.03-1.67 | 0.03 |
| Junctional zone size (mm) | 5.54±1.84 | 6.51±2.80 | 0.93 | 0.62-1.83 | 0.84 |
| Mean myometrium (mm) | 26.16±16.46 | 23.82±11.86 | 1.54 | 0.58-2.43 | 0.23 |
| Junctional zone/myometrium ratio | 0.32±0.14 | 0.41±0.12 | 0.65 | 0.34-3.45 | 0.47 |

| Table S4 Univariate Analyses of clinical characteristics related with asymptomatic | | | | | |
| --- | --- | --- | --- | --- | --- |
| Clinical Variable | Positive (133) | negative (272) | OR | 95%CI | P-value |
| age, y | 37.69±3.25 | 44.51±4.91 | 0.74 | 0.56-0.81 | 0.01 |
| height, cm | 160.83±3.84 | 159.83±4.81 | 1.23 | 0.78-1.59 | 0.24 |
| weight, kg | 59.41±2.81 | 60.67± 2.86 | 1.16 | 0.83-1.57 | 0.57 |
| BMI, kg/m2 | 21.81±3.10 | 23.14±2.84 | 1.27 | 0.98-1.89 | 0.03 |
| Gravidity | 1.34±0.33 | 0.73±0.57 | 1.43 | 0.64-1.75 | 0.02 |
| Parity | 1.78±0.63 | 1.64±0.24 | 1.39 | 0.84-1.62 | 0.52 |
| mean age at menarche, y | 14.76±0.79 | 13.38±0.61 | 1.33 | 0.79-2.18 | 0.56 |
| mean duration cycle, d | 31.71±7.37 | 30.45±6.45 | 1.65 | 0.85-2.53 | 0.26 |
| mean length of menstruations, d | 6.22±1.77 | 8.25±1.15 | 1.47 | 0.87-1.72 | 0.04 |
| regular menstrual cycle (n, %) | 117, 87.97 | 245, 90.07 | 1.25 | 0.67-2.97 | 0.42 |
| Endometriotic cyst (n, %) | 119, 43.75 | 78, 58.65 | 1.65 | 0.62-0.82 | 0.02 |
| Deep infiltrating endometriosis | 53, 39.85 | 101, 37.13 | 1.41 | 0.67-2.62 | 0.23 |
| Uterine myoma (n, %) | 58, 43.61 | 131, 48.16 | 0.85 | 0.73-3.57 | 0.34 |
| history of miscarriage (n, %) | 34 25.56 | 64, 23.89 | 0.91 | 0.65-2.34 | 0.51 |
| previous uterine surgery (n, %)) | 60, 45.11 | 101, 37.13 | 1.54 | 0.73-1.56 | 0.27 |
| CA125 | 61.56±23.21 | 72.16±15.58 | 1.36 | 0.78-1.89 | 0.36 |
| Junctional zone size (mm) | 5.79±3.21 | 6.55±2.81 | 0.67 | 0.58-0.78 | 0.02 |
| Mean myometrium (mm) | 23.92±3.21 | 28.86±5.31 | 1.79 | 0.84-1.98 | 0.18 |
| Junctional zone/myometrium ratio | 0.29±0.034 | 0.41±0.053 | 1.13 | 0.75-2.78 | 0.17 |

Appendix E2 clinical score calculation formula

Pain label= -2.2256+0.4420*age+0.3346*DIE+ 0.0160* CA125

AUB label= -4.3956+0.3217*BMI+0.2346* regular menstrual cycle+0.2458 *miscarriage

Infertility label=-3,4521+0.3421*age+ 0.2214*BMI+0.1256* regular menstrual cycle+0.3255*Endometriotic cyst

Asymptomatic label=3.6543-0.3245* Age-0.2432* Gravity-0.4521* Endometriotic cyst-0.5436*Junctional zone size

Figure S5 Decision curve analysis and calibration curve of nomogram for symptom prediction in the training and test cohort


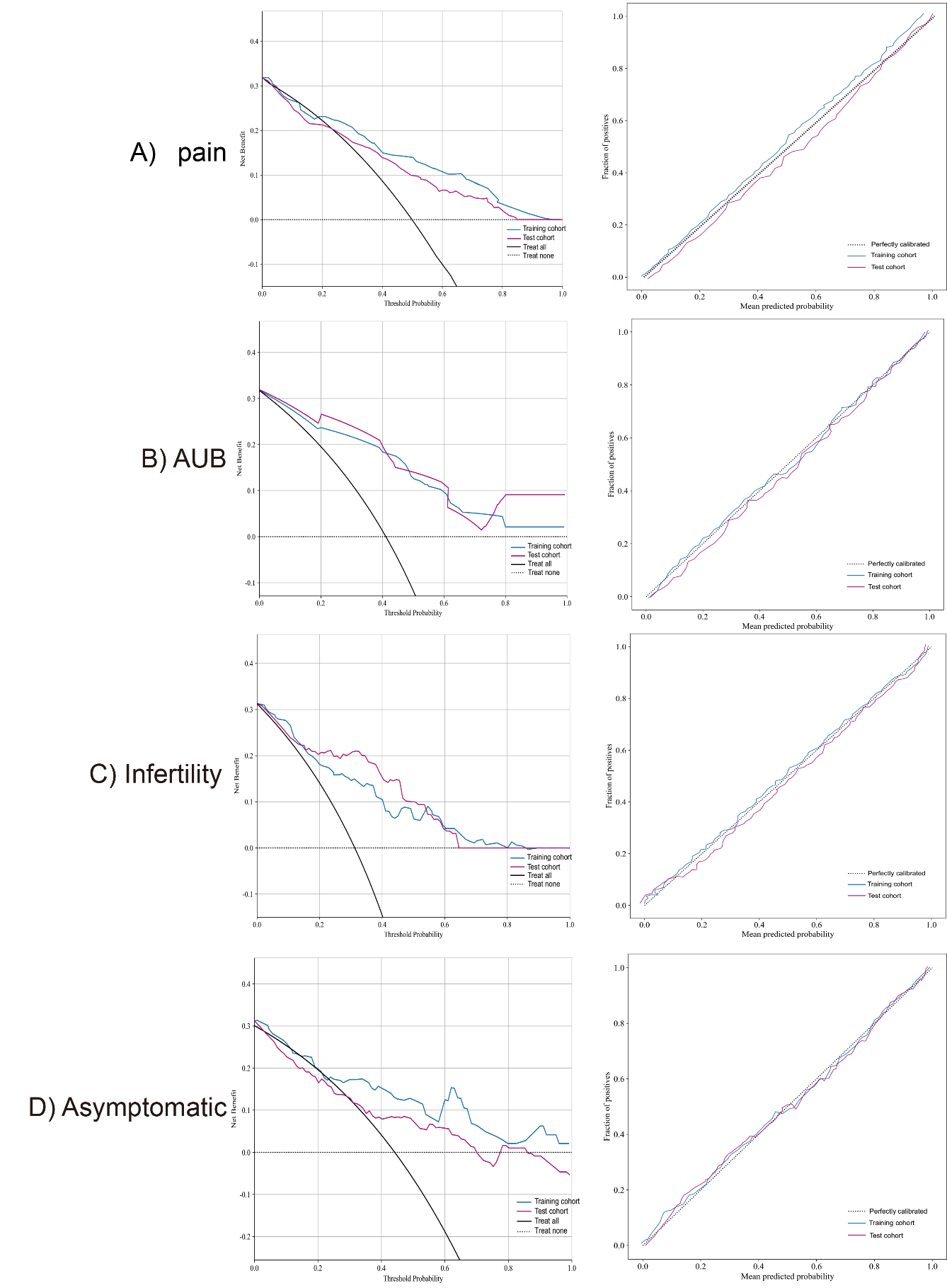

Supplement: Supplemental Material [file IANN_A_2534521_SM4803.zip › suppl_data/All Supplementary table.docx]
