# Supplementary material for: Development and validation of MRI-based radiomics model for clinical symptom stratification of extrinsic adenomyosis
Source: Ann Med. 2025 Jul 25;57(1):2534521. doi: 10.1080/07853890.2025.2534521 (PMC12302386; doi:10.1080/07853890.2025.2534521)
Supplement: Supplemental Material [file IANN_A_2534521_SM4803.zip › suppl_data/Supplementary figure legends.docx]

Supplementary figure legends

Figure S1 Correlation heatmap of radiomic features for pain symptom

Figure S2 Correlation heatmap of radiomic features for AUB symptom

Figure S3 Correlation heatmap of radiomic features for infertility symptom

Figure S4 Correlation heatmap of radiomic features for asymptomatic patients

Appendix E1 radscore calculation formula

Table S1 Univariate Analyses of clinical characteristics related with pain. OR Odds ratio, 95% CI 95% confidence interval

Table S2 Univariate Analyses of clinical characteristics related with AUB. OR Odds ratio, 95% CI 95% confidence interval

Table S3 Univariate Analyses of clinical characteristics related with infertility. OR Odds ratio, 95% CI 95% confidence interval

Table S4 Univariate Analyses of clinical characteristics related with asymptomatic. OR Odds ratio, 95% CI 95% confidence interval

Appendix E2 clinical score calculation formula

Figure S5 Decision curve analysis and calibration curve of nomogram for symptom prediction in the training and test cohort. (A) pain, (B)AUB, (C) Infertility and (D) Asymptomatic
