# Supplementary figures and images for: Development and validation of MRI-based radiomics model for clinical symptom stratification of extrinsic adenomyosis
Source: Ann Med. 2025 Jul 25;57(1):2534521. doi: 10.1080/07853890.2025.2534521 (PMC12302386; doi:10.1080/07853890.2025.2534521)

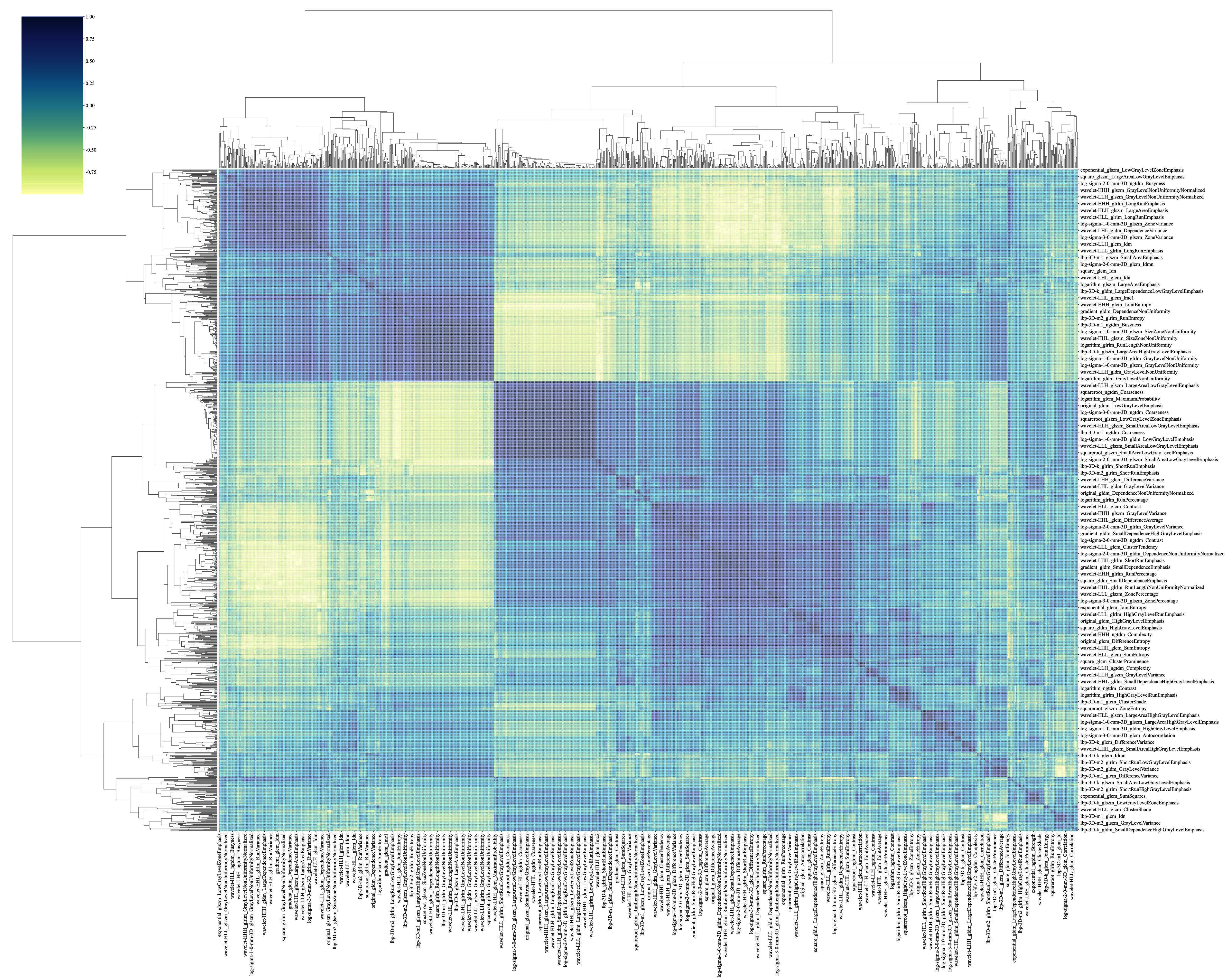

Supplement: Supplemental Material [file IANN_A_2534521_SM4803.zip › suppl_data/Figure S2 heatmap aub.tif]

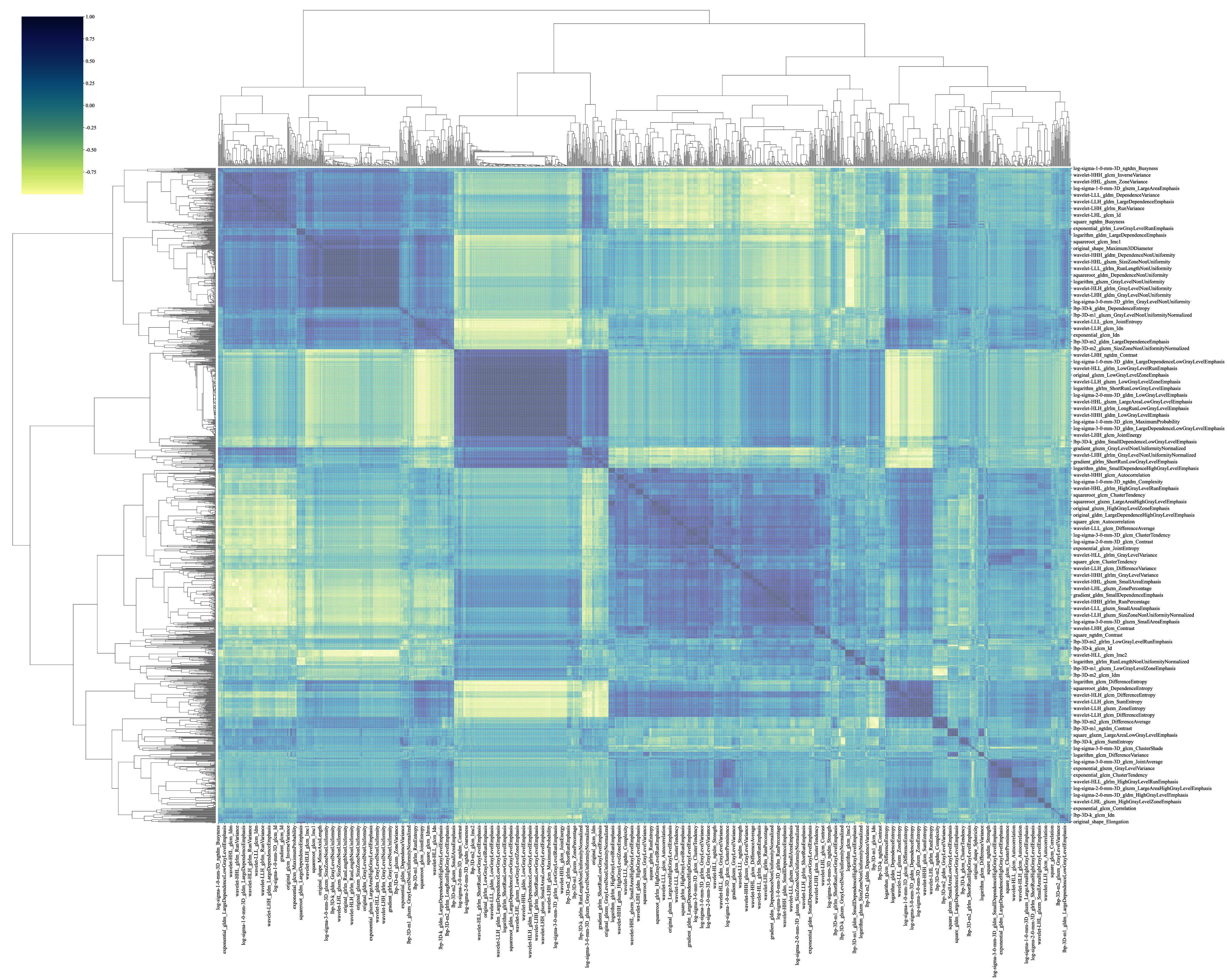

Supplement: Supplemental Material [file IANN_A_2534521_SM4803.zip › suppl_data/FIgure S3 heatmap infertility.tif]

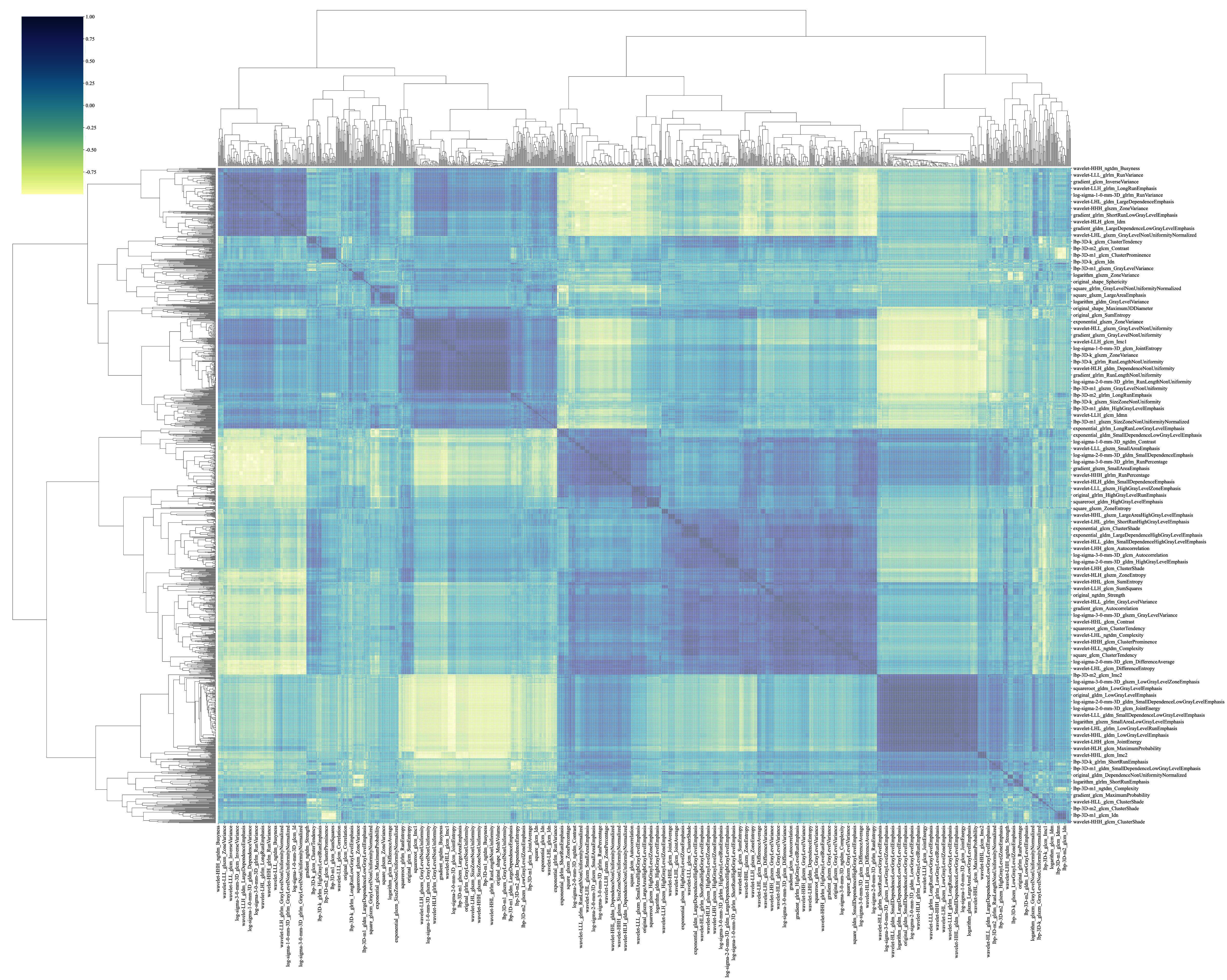

Supplement: Supplemental Material [file IANN_A_2534521_SM4803.zip › suppl_data/Figure S4 heatmap asymptomatic.tif]
